# Time Series of Counts under Censoring: A Bayesian Approach

**DOI:** 10.3390/e25040549

**Published:** 2023-03-23

**Authors:** Isabel Silva, Maria Eduarda Silva, Isabel Pereira, Brendan McCabe

**Affiliations:** 1 Faculdade de Engenharia, Universidade do Porto, CIDMA, 4200-465 Porto, Portugal; 2 Faculdade de Economia, Universidade do Porto, LIADD-INESC TEC, 4200-464 Porto, Portugal; 3 Departamento de Matemática, Universidade de Aveiro, CIDMA, 3810-193 Aveiro, Portugal; 4 School of Management, University of Liverpool, Liverpool L69 3BX, UK

**Keywords:** Bayesian estimation, censored time series, convolution closed infinitely divisible, Poisson INAR(1) model

## Abstract

Censored data are frequently found in diverse fields including environmental monitoring, medicine, economics and social sciences. Censoring occurs when observations are available only for a restricted range, e.g., due to a detection limit. Ignoring censoring produces biased estimates and unreliable statistical inference. The aim of this work is to contribute to the modelling of time series of counts under censoring using convolution closed infinitely divisible (CCID) models. The emphasis is on estimation and inference problems, using Bayesian approaches with Approximate Bayesian Computation (ABC) and Gibbs sampler with Data Augmentation (GDA) algorithms.

## 1. Introduction

Observations collected over time or space are usually correlated rather than independent. Time series are often observed with data irregularities such as missing values or detection limits. For instance, a monitoring device may have a technical detection limit and it records the limit value when the true value exceeds/precedes the detection limit. Such data is called censored (type 1) data and are common in environmental monitoring, physical sciences, business and economics. In particular, in the context of time series of counts, censored data arise in call centers. In fact, the demand measured by the number of calls is limited by the number of operators. When the number of calls is higher than the number of operators the data is right censored and the call center incurs under-staffing and poor service to the costumers.

The main consequence of neglecting censoring in the time series analysis is the loss of information that is reflected in biased and inconsistent estimators and altered serial correlation. These consequences can be summarized as problems in inference that lead to model misspecification, biased parameter estimation, and poor forecasts.

These problems have been solved in regression settings (i.i.d.) and partially solved for Gaussian time series (see for instance [1,2,3,4,5,6,7]). However, the problem of modelling time series under censoring in the context of time series of counts has, as yet, received little attention in the literature even though its relevance for inference. Count time series occur in many areas such as telecommunications, actuarial science, epidemiology, hydrology and environmental studies where the modelling of censored data may be invaluable in risk assessment.

In the context of time series of counts, Ref. [8] deal with correlated under-reported data through INAR(1)-hidden Markov chain models. A naïve method of parameter estimation was proposed, jointly with the maximum likelihood method based on a revised version of the forward algorithm. Additionally, Ref. [9] propose a random-censoring Poisson model for under-reported data, which accounts for the uncertainty about both the count and the data reporting processes.

Here, the problem of modelling count data under censoring is considered under a Bayesian perspective. In this paper, we consider a general class of convolution closed infinitely divisible (CCID) models as proposed by [10].

We investigate two natural approaches to analyse censored convolution closed infinitely divisible models of first order, CCID(1), using the Bayesian framework: the Approximate Bayesian Computation (ABC) methodology and the Gibbs sampler with Data Augmentation (GDA).

Since the CCID(1) under censoring presents an intractable likelihood, we resort to the Approximate Bayesian Computation methodology for estimating the model parameters. The presupposed model is simulated by using sample parameters taken from the prior distribution, then a distance between the simulated dataset and the observations is computed and when the simulated dataset is *very close* to the observed, the corresponding parameter samples are accepted as part of the posterior.

In addition, a widely used strategy to deal with censored data is to fill in censored data in order to create a data-augmented (complete) dataset. When the data-augmented posterior and the conditional pdf of the latent process are both available in a tractable form, the Gibbs sampler allows us to sample from the posterior distribution of the parameters of the complete dataset. This methodology is called Gibbs sampler with Data Augmentation (GDA). Here, a modified GDA, in which the data augmentation is achieved by multiple sampling of the latent variables from the truncated conditional distributions (GDA-MMS), is adopted.

**Figure 1:**
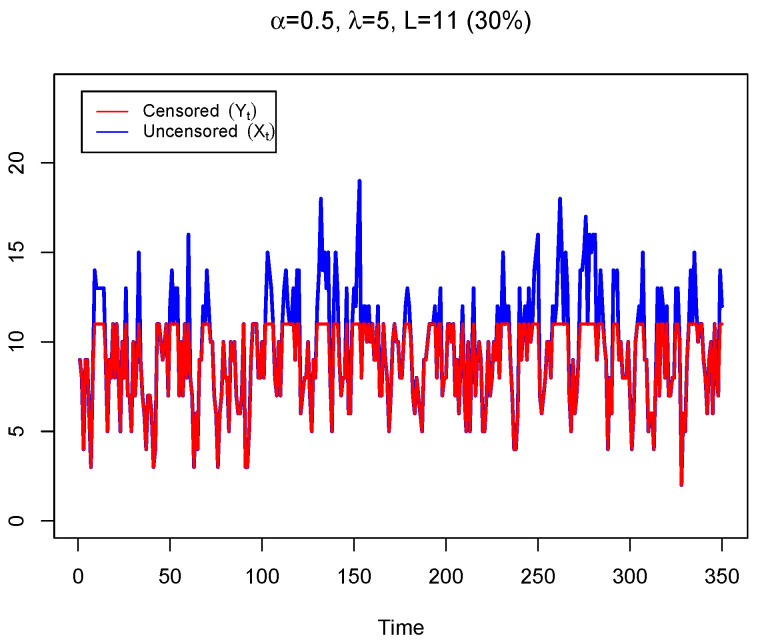
Synthetic dataset with n=350 observations generated from a PoINAR(1) process with parameters α=0.5 and λ=5 ($X_{t},$ blue line) and the respective right censored dataset ($Y_{t},$ red line), at L=11.

The Poisson integer-valued autoregressive models of first-order, PoINAR(1), is one of the most popular classes of CCID models. It was proposed by [11,12] and extensively studied in the literature and applied to many real-world problems because of its ease of interpretation. To motivate the proposed approaches, we present in Figure 1 a synthetic dataset with n=350 observations generated from a PoINAR(1) process with parameters α=0.5 and λ=5 ($X_{t},$ blue line) and the respective right-censored dataset ($Y_{t},$ red line), at L=11, corresponding to 30% of censoring. If we disregard the censoring, the estimates for the parameters (assuming an PoINAR(1) model without censoring) present a strong bias. For instance, in the frequentist framework, the conditional maximum likelihood estimates are ${\hat{\alpha }}_{CML}=0.6174$ and ${\hat{\lambda }}_{CML}=3.4078,$ while in the Bayesian framework, the Gibbs sampler gives ${\hat{\alpha }}_{Bayes}=0.6242$ and ${\hat{\lambda }}_{Bayes}=3.3297.$ On the other hand, if we assume a PoINAR(1) model under censoring, the parameter estimates given by the proposed approaches described in this work are, respectively, ${\hat{\alpha }}_{ABC}=0.4623$ and ${\hat{\lambda }}_{ABC}=5.2259,$ and ${\hat{\alpha }}_{GDA}=0.4834$ and ${\hat{\lambda }}_{GDA}=4.9073.$ Therefore, it is important to consider the censoring in data in order to avoid some inference issues that lead to a poor time series analysis.

The remainder of this work is organized as follows. Section 2 presents a general class of convolution closed infinitely divisible (CCID) models under censoring. Two Bayesian approaches proposed to estimate the parameters of the censored CCID(1) model are described in Section 3. The proposed methodologies are illustrated and compared with synthetic data in Section 4. Finally, Section 5 concludes the paper.

## 2. A Model for Time Series of Counts under Censoring

This section introduces a class of models adequate for censored time series of counts based on the convolution closed infinitely divisible (CCID) models as proposed by [10].

### 2.1. Convolution Closed Models for Count Time Series

First we introduce some notation. Consider a random variable *X* with a distribution $F_{\mu },$ μ>0, belonging to the convolution closed infinitely divisible (CCID) parametric family [10]. This means, in particular, that the distribution $F_{\mu }$ is closed under convolution, $F_{\mu_{1}}*F_{\mu_{2}}=F_{\mu_{1}+\mu_{2}},$ where * is the convolution operator. Let R(·) denote a random operator on *X* such that $R\left(X\right)∼F_{\alpha \mu },$ 0<α<1 and the conditional distribution of R(X) given X=x is $G_{\alpha \mu ,(1-\alpha )\mu ,x},$ $R\left(X\right)|X=x∼G_{\alpha \mu ,(1-\alpha )\mu ,x}.$ As an example, consider a Poisson random variable, X∼Po(μ) and a binomial thinning operation, $R\left(X\right)=\alpha \circ X=\sum_{i=1}^{X}\xi_{i},$ $\xi_{i}{∼}^{iid}Ber\left(\alpha \right).$ Then $F_{\mu }$ is the Poisson distribution with parameter μ, R(X)∼Po(αμ) and R(X)|X=x∼Bi(x,α), $G_{\alpha \mu ,(1-\alpha )\mu ,x}$ is the Binomial distribution with parameters *x* and α.

A stationary time series, $\left\{X_{t};t=0,\pm 1,\pm 2,\ldots \right\}$ with margin $F_{\mu },$ $X_{t}∼F_{\mu },$ is called a convolution closed infinitely divisible process of order 1, CCID(1), if it satisfies the following equation

(1) $$X_{t}=R_{t}\left(X_{t-1}\right)+e_{t},$$

where the innovations $e_{t}$ are independently and identically distributed (i.i.d.) with distribution $F_{(1-\alpha )\mu }$ and $\left\{R_{t}\left(\cdot \right):t=0,\pm 1,\pm 2,\ldots \right\}$ are independent replications of the random operator R(·) [10]. Note that the above construction leads to time series with the same marginal distribution as that of the innovations.

Model (1) encompasses many AR(1) models proposed in the literature for integer valued time series. In particular, the Poisson INAR(1), PoINAR(1), the negative binomial INAR(1), NBINAR(1), and the generalised Poisson INAR(1), GPINAR(1) [13], summarized in Table 1 ( marginal distribution, random operation and its pmf g(·|·), set of parameters θ), have been widely used in the literature to model time series of counts, see *inter alia* [14,15], among others.

If one chooses $F_{(1-\alpha )\mu }$ as Poisson ((1−α)μ), and the random operation as the usual binomial thinning operation (based on underlying Bernoulli random variables) $R_{t}\left(X_{t-1}\right)=\alpha \circ X_{t-1}=\sum_{i=1}^{X_{t-1}}\xi_{ti},$ $\xi_{ti}{∼}^{iid}Ber\left(\alpha \right),$ then $F_{\mu }$ is Poisson (μ) and the Poisson integer-valued autoregressive model, PoINAR(1), as proposed by [11,12], is recovered with the familiar representation

(2) $$X_{t}=\alpha \circ X_{t-1}+\epsilon_{t}.$$

Since model (1) is Markovian [10], given a time series $x=(X_{1},\ldots ,X_{n}),$ the conditional likelihood is as follows

(3) $$L\left(\theta \right)=\prod_{t=2}^{n}f_{X_{t}|X_{t-1}}\left(x_{t}|x_{t-1}\right),$$

with

(4) $$f_{X_{t}|X_{t-1}}\left(k|l\right)=P\left(X_{t}=k|X_{t-1}=l\right)=\sum_{j=0}^{\min\{k,l\}}g\left(j|l\right)P\left(e_{t}=k-j\right).$$

### 2.2. Modelling Censoring in CCID(1) Time Series

Given a model as in (1), a basic question is whether it properly describes all the observations of a given time series, or whether some observations have been affected by censoring. Here, we describe a model for dealing with censored observations in CCID(1) processes and study some of its properties.

Exogenous censoring can be modelled assuming (1) as a latent process and $Y_{t}=\min\left\{L,X_{t}\right\}$ as the observed process, where *L* is a constant that is assumed to be known. For simplicity of exposition we assume exogenous right censoring but all the results are easily extended to left-censoring or interval censoring. Hence, for right exogenous censoring

(5) $$\begin{array}{ccc} Y_{t} & = & \min\left\{X_{t},L\right\}=\left\{\begin{array}{cc} X_{t}, & \mathrm{if}X_{t}<L,\\ L, & \mathrm{if}X_{t}\geq L, \end{array}\right.\\ X_{t} & = & R_{t}\left(X_{t-1}\right)+e_{t}. \end{array}$$

Although $X_{t},$ a CCID(1) process is Markovian, the exogenous censoring implies that $Y_{t}$ is not Markovian because $Y_{t}$ depends on $X_{t}$ and L. Furthermore, $Y_{t}$ is not CLAR (Conditionally Linear AutoRegressive). In fact,

$$\begin{array}{ccc} E\left[Y_{t}|Y_{t-1}=y_{t-1}\right] & = & E\left[Y_{t}|Y_{t-1}=y_{t-1}\right]I_{\left\{y_{t-1}<L\right\}}+E\left[Y_{t}|Y_{t-1}=L\right]I_{\left\{y_{t-1}=L\right\}}\\ & = & \left(E\left[X_{t}|X_{t-1}=y_{t-1}\right]-\sum_{j=0}^{+\infty }jP\left[X_{t}=L+j|X_{t-1}=y_{t-1}\right]\right)I_{\left\{y_{t-1}<L\right\}}\\ & & +\left(E\left[X_{t}|X_{t-1}\geq L\right]-\sum_{j=0}^{+\infty }jP\left[X_{t}=L+j|X_{t-1}\geq L\right]\right)I_{\left\{y_{t-1}=L\right\}} \end{array}$$

The authors Zeger and Brookmeyer [1] established a procedure to obtain the likelihood of an observed time series under censoring, $y=(Y_{1},\ldots ,Y_{n}),$ which becomes infeasible when the proportion of censoring is large. To overcome this issue, this work considers a Bayesian approach.

## 3. Bayesian Modelling

The Bayesian approach to the inference of an unknown parameter vector θ is based on the posterior distribution π(θ|y), defined as

π(θ|y)∝L(y|θ)π(θ),

where L(y|θ) is the likelihood function of the observed data y and π(θ) is the prior distribution of the model parameters.

When the likelihood is computationally prohibitive or even impossible to handle, but it is feasible to simulate samples from the model (bypass the likelihood evaluation), as is the case of censored CCID(1) processes, Approximate Bayesian Computation (ABC) algorithms are an alternative. This methodology accepts the parameter draws that produce a match between the observed and the simulated sample, depending on a set of summary statistics, a chosen distance and a selected tolerance. The accepted parameters are then used to estimate (an approximation of) the posterior distribution (conditioned on the summary statistics that afforded the match).

On the other hand, the idea of imputation arises naturally in the context of censored data. The Gibbs sampler with Data Augmentation (GDA) allows us to obtain an augmented dataset from the censored data by using a modified version of the Gibbs sampler, which samples not only the parameters of the model from its complete conditional but also the censored observations. The usual inference procedures may then be applied to the augmented data set.

### 3.1. Approximate Bayesian Computation

Approximate Bayesian Computation (ABC) is based on an acceptance–rejection algorithm. ABC is used to compute a draw from an approximation of the posterior distributions, based on simulated data obtained from the assumed model in situations where its likelihood function is intractable or numerically difficult to handle. Summary statistics from the synthetic data are compared with the corresponding statistics from the observed data and a parameter draw is retained when there is a *match* (in some sense) between the simulated sample and the observed time series observation.

Recently, Ref. [16] provided the asymptotic results pertaining to the ABC posterior, such as Bayesian consistency and asymptotic distribution of the posterior mean.

Let $y^{0}=\left(Y_{1}^{0},\ldots ,Y_{n}^{0}\right)$ be the fixed (observed) data and η(·) the model from which the data is generated. The most basic *approximate acceptance/rejection algorithm*, based on the works of [17,18], is as follows:

1. draw a value θ from the prior distribution, π(θ),
2. simulate a sample $y=(Y_{1},\ldots ,Y_{n})$ from the model η(.|θ),
3. accept θ if $d(S\left(y\right),S_{0}))\leq \delta$ for some distance measure d(.,.) and some non-negative tolerance value δ, where S(·) is a summary statistic and $S_{0}=S\left(y^{0}\right)$ is a fixed value.

It is well known that, if we use a proper distance measure, then as δ tends to zero, the distribution of the accepted values tends to the posterior distribution of the parameter given the data. When the summary statistics are sufficient for the parameter, then the distribution of the accepted values tends to the true posterior as δ tends to zero, assuming a proper distance measure on the space of sufficient statistics. The latent structure of the thinning operator means that the reduction to a sufficient set of statistics of dimension smaller than the sample size is not feasible and, therefore, informative summary statistics are often used [19].

In this work, given the characteristics of the data under study to compare the observed data ($y^{0}$) and the synthetic (simulated ) data (y), we consider two distinctive characteristics of CCID(1) time series which are affected by the censoring: (i) the empirical marginal distribution and (ii) lag 1 auto-correlation.

To measure the similarity between the empirical marginal distributions the Kullback-Leibler (Note that Kullback-Leibler distance measures the dissimilarity between two probability distributions.) distance is calculated as

(6) $$S_{1}\left(y\right)=d_{KL}\left({\hat{p}}^{0},\hat{p}\right)=\sum_{j}\ln\left(\frac{{\hat{p}}_{j}^{0}}{{\hat{p}}_{j}}\right){\hat{p}}_{j}^{0},$$

where ${\hat{p}}_{j}^{0}$ and ${\hat{p}}_{j}$ denote the empirical marginal distribution of the observed time series and that of the simulated time series, respectively, estimated by the corresponding sample proportions, $\left({\hat{p}}_{j}^{0}=\frac{1}{n}\sum_{j=1}^{n}I_{\left\{Y_{j}^{0}=j\right\}}\;\mathrm{and}\;{\hat{p}}_{j}=\frac{1}{n}\sum_{j=1}^{n}I_{\left\{Y_{j}=j\right\}}\right).$ Whenever ${\hat{p}}_{j}^{0}$ is zero, the contribution of the *j*th term is interpreted as zero because $\lim_{p\to 0}p\ln(p)=0$.

On the other hand, lag 1 sample autocorrelations, $S_{2}\left(y^{0}\right)={\hat{\rho }}_{Y^{0}}\left(1\right)$ and $S_{2}\left(y\right)={\hat{\rho }}_{Y}\left(1\right),$ are compared by their squared difference.

Additionally, we estimated the censoring rates, $S_{3}\left(y^{0}\right)=\frac{1}{n}\sum_{t=1}^{n}I_{\left\{y_{t}^{0}=L\right\}}$ and $S_{3}\left(y\right)=\frac{1}{n}\sum_{t=1}^{n}I_{\left\{Y_{t}=L\right\}},$ which are also compared by their squared difference.

Thus, for each set of parameters, $\theta^{\left(k\right)}$, a time series $x^{\left(k\right)}$ is generated from the model CCID(1) and right censored at *L*, yielding $y^{\left(k\right)}=\left(Y_{1}^{\left(k\right)},\ldots ,Y_{n}^{\left(k\right)}\right)$ and the above statistics, $S_{1}\left(y^{\left(k\right)}\right),$ $S_{2}\left(y^{\left(k\right)}\right)$ and $S_{3}\left(y^{\left(k\right)}\right)$ are computed. Combining these statistics in a metric leading to the choice of the parameters θ requires scaling. Thus, we propose the following metric

(7) $$d_{S}^{\left(k\right)}=\frac{S_{1}{\left(y^{\left(k\right)}\right)}^{2}}{V(S_{1}\left(y\right))}+\sum_{i=2}^{3}\frac{{\left[S_{i}\left(y^{0}\right)-S_{i}\left(y^{\left(k\right)}\right)\right]}^{2}}{V(S_{i}\left(y^{0}\right)-S_{i}\left(y\right))}$$

where $S_{i}\left(y^{0}\right)$ and $S_{i}\left(y^{\left(k\right)}\right)$ are the ith statistics obtained respectively from the observed and kth simulated data and $V(S_{1}\left(y\right))$ and $V(S_{i}\left(y^{0}\right)-S_{i}\left(y\right))$ are the corresponding sample variances across the replications.

In summary, we propose Algorithm 1 for ABC approach based on [20]:
**Algorithm 1** ABC for censored CCID(1)For k=1,…,N     Sample $\theta^{\left(k\right)}$ from the prior distribution π(θ)     Generate a time series with *n* observations, $x^{\left(k\right)}$ from the CCID(1) model     Right truncate at *L* $x^{\left(k\right)}$ to obtain the simulated data $y^{\left(k\right)}$     Compute $S_{1}\left(y^{\left(k\right)}\right),$ $S_{2}\left(y^{\left(k\right)}\right)$ and $S_{3}\left(y^{\left(k\right)}\right)$Compute $d_{S}^{\left(k\right)}=\frac{S_{1}{\left(y^{\left(k\right)}\right)}^{2}}{V(S_{1}\left(y\right))}+\sum_{i=2}^{3}\frac{{\left[S_{i}\left(y^{0}\right)-S_{i}\left(y^{\left(k\right)}\right)\right]}^{2}}{V(S_{i}\left(y^{0}\right)-S_{i}\left(y\right))}$, k=1,…,NSelect the values $\theta^{\left(k\right)}$ corresponding to the 0.1% quantile of $d_{S}^{\left(k\right)},$ k=1,…,N

Implementation issues regarding the prior distributions and the number of draws *N* for the CensPoINAR(1) model are addressed in Section 3.3 and Section 4.1.

### 3.2. Gibbs Sampler with Data Augmentation

Gibbs sampling is a Markov chain Monte Carlo (MCMC) algorithm that can generate samples of the posterior distribution from their full conditional distributions [21]. When the data are under censoring or there are missing values, both cases leading to an incomplete data set, Ref. [22] proposed to combine the Gibbs sampler with data augmentation. This methodology implies imputing the censored (or missing) data, thus obtaining a complete dataset, and then dealing with the posterior of the complete data through the iterative Gibbs sampler. Therefore, the Gibbs sampler is modified in order to sample not only the parameters of the model from their complete conditionals but also the censored observations, obtaining an augmented (complete) dataset $z=(z_{1},\ldots ,z_{n})$ where

(8) $$z_{t}=\left\{\begin{array}{cc} Y_{t}, & \mathrm{if}\;Y_{t}<L\\ z_{t}∼F_{\mu }\left(x|x\geq L\right), & \mathrm{if}\;Y_{t}=L \end{array}\right.$$

where $F_{\mu }\left(x|x\geq L\right)$ is the truncated marginal distribution of the CCID(1) model with support in [L,+∞[. Furthermore, we consider a modified sampling procedure for the imputation, designated as Mean of Multiple Simulation (MMS), proposed by [23] consisting in sampling from $F_{\mu }\left(x|x\geq L\right)$ multiple times, say m, and then imputing with the (nearest integer value) median of the *m* samples. This procedure is designated by **GDA-MMS**.

The augmented dataset can be considered as a CCID(1) time series and with a conditional likelihood function given by Equation (3). The posterior distribution of θ is given by

(9) p(θ|z)∝L(z|θ)π(θ)

where π(θ) is the prior distribution of the parameters. In CCID(1) models the complexity of p(θ|z) requires resorting to Markov Chain Monte Carlo (MCMC) techniques for sampling from the full conditional distributions. The procedure is summarized in Algorithm 2 and detailed for the CensPoINAR(1) case in Section 3.3 and Section 4.1.
**Algorithm 2** GDA-MMS for censored CCID(1)Initialize with $y=(Y_{1},\ldots ,Y_{n})$, $\theta^{\left(0\right)}=\left(\theta_{1}^{\left(0\right)},\ldots ,\theta_{p}^{\left(0\right)}\right)$, L∈R, and n,m,N∈NSet $z^{\left(0\right)}=y$For k=1,…,N     Sample $\theta_{i}^{\left(k\right)}∼\pi (\theta_{i}\left|\theta_{\left(-i\right)}^{\left(k-1\right)},z^{\left(k-1\right)}\right)$ ($x_{\left(-i\right)}$ represents the vector x with the *i*th elementremoved.), i=1,…,p     For t=1,…,n        If $Y_{t}=L$            For j=1,…,m               Sample $z_{t}^{\left(j\right)}∼F(x\left|\theta^{\left(k\right)},x\geq L\right)$            $z_{t}^{\left(k\right)}:=\frac{1}{m}\sum_{j=1}^{m}z_{t}^{\left(j\right)}$       Else           $z_{t}^{\left(k\right)}:=Y_{t}$Return $\theta ={\left[\theta^{\left(1\right)},\ldots ,\theta^{\left(N\right)}\right]}^{\prime }$ and $z^{\left(N\right)}$.

### 3.3. The Particular Case of CensPoINAR(1)

This section details the ABC and GDA-MMS procedures to estimate a censored CCID(1) with the binomial thinning operation and Poisson marginal distribution, the censored Poisson INAR(1), CensPoINAR(1), model.

Consider the censored observations $y=(Y_{1},\ldots ,Y_{n})$ from a PoINAR(1) time series $x=(X_{1},\ldots ,X_{n})$ defined as

(10) $$\begin{array}{ccc} Y_{t} & = & \min\left\{X_{t},L\right\}=\left\{\begin{array}{cc} X_{t}, & \mathrm{if}\;X_{t}<L\\ L, & \mathrm{if}\;X_{t}\geq L \end{array}\right.\\ X_{t} & = & \alpha \circ X_{t-1}+e_{t}, \end{array}$$

with $\alpha \circ X_{t-1}=\sum_{i=1}^{X_{t-1}}\xi_{ti},$ $\xi_{ti}{∼}^{iid}Ber\left(\alpha \right),$ $e_{t}∼Po\left(\lambda \right)$ and $X_{t}∼Po\left(\frac{\lambda }{1-\alpha }\right).$ Then θ=(α,λ) and given x, the conditional likelihood function is given by

(11) L(θ)=∏t=2nfXt|Xt−1(xt|xt−1)=∏t=2n∑j=0min{xt,xt−1}xt−1jαj(1−α)(xt−1−j)e−λλxt−j(xt−j)!.

Under a Bayesian approach, we need a prior distribution for θ. In the absence of prior information, we use weakly informative prior distributions for θ detailed below.

#### 3.3.1. ABC for Censored PoINAR(1)

The ABC procedure described in Algorithm 1 is now implemented for the censored PoINAR(1). For the parameter 0<α<1, we choose a non-informative prior U(0,1), while for the positive parameter λ, we choose a non-informative U(0,10). The former allows us to explore all the support space for α. The choice of U(0,10) as a prior for λ>0 allows us to explore a restricted support for the parameter that is in accordance with small counts.

#### 3.3.2. GDA-MMS for Censored PoINAR(1)

**Figure 2:**
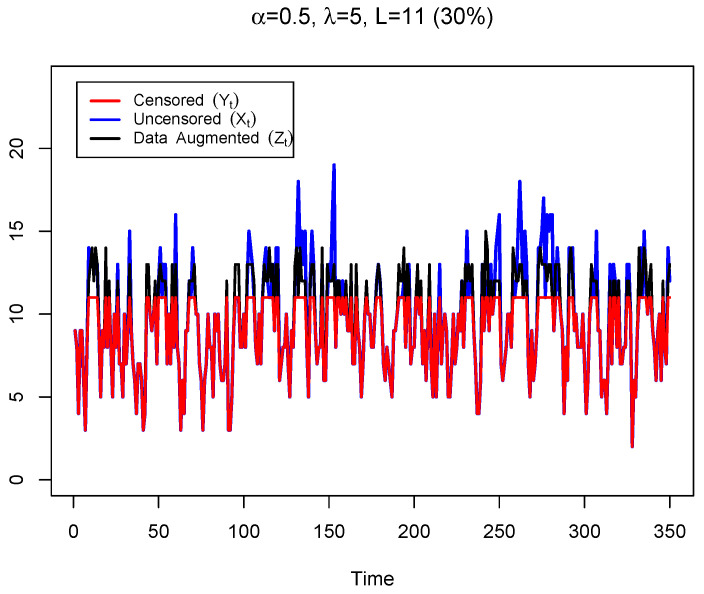
Synthetic dataset with n=350 observations generated from a PoINAR(1) process with parameters α=0.5 and λ=5 ($X_{t},$ blue line), the respective right-censored dataset ($Y_{t},$ red line), at L=11, and an example of data augmentation ($Z_{t},$ black line).

Under the GDA-MMS approach, we first need to obtain a complete data set $z=(z_{1},\ldots ,z_{n})$ by imputing the censored observations, see (8). In this work, we draw m=10 replicates of the right truncated at *L* Poisson distribution with parameter $\frac{\lambda }{1-\alpha },$ $w_{i}∼\mathrm{Po}\left(\frac{\lambda }{1-\alpha }\right)\times I_{\left(w_{i}\geq L\right)}$ and set $z_{t}=\left\lceil median\left(w\right)\right\rceil$ (⌈c⌉ represents the integer ceiling of *c*), $w=(w_{1},\ldots ,w_{m}),$ if $Y_{t}=L.$ Figure 2 shows an augmented dataset ($Z_{t},$ black line) from the synthetic data presented in Figure 1.

As remarked above, given the complexity of the posterior distribution, Markov Chain Monte Carlo techniques are required for sampling from the full conditional distributions. Thus, the Adaptive Rejection Metropolis Sampling (ARMS) is used inside the Gibbs sampler [24]. Also in this approach, in the absence of prior information, we use weakly informative prior distributions for (α,λ). Thus, for the parameter 0<α<1, we choose a non-informative beta prior, conjugate of the binomial distribution, with parameters (a,b), while for the positive parameter μ, we choose a non-informative Gamma (shape, rate) prior, conjugate of the Poisson distribution, with parameters (c,d). The full conditional of λ is given by

(12) $$p\left(\lambda |\alpha ,z\right)=\frac{p(\lambda ,\alpha |z)}{p(\alpha |z)}\propto \exp\left[-\left(d+\left(n-1\right)\right)\lambda \right]\lambda^{c-1}\prod_{t=2}^{n}\sum_{i=0}^{\min\{z_{t},z_{t-1}\}}C\left(t,i\right)\lambda^{(z_{t})-i},$$

where

C(t,i)=1((zt)−i)!zt−1iαi(1−α)(zt−1)−iandλ>0.

The full conditional distribution of α is given by

(13) $$p\left(\alpha |\lambda ,z\right)=\frac{p(\lambda ,\alpha |z)}{p(\lambda |z)}\propto \alpha^{a-1}{\left(1-\alpha \right)}^{b-1}\prod_{t=2}^{n}\sum_{i=0}^{\min\{z_{t},z_{t-1}\}}K\left(t,i\right)\alpha^{i}{\left(1-\alpha \right)}^{(z_{t-1})-i},$$

where

K(t,i)=λ(zt)−i((zt)−i)!Xt−1i0<α<1.

The parameters α and λ are computed as the posterior mean.

The GDA-MMS procedure to estimate a censored PoINAR(1) process is detailed in Algorithm 3.
**Algorithm 3** GDA-MMS for CensPoINAR(1)Initialize with y, $\theta^{\left(0\right)}=\left(\alpha^{\left(0\right)},\lambda^{\left(0\right)}\right)$, L∈R, and N,m∈NSet $z^{\left(0\right)}=y$For k=1,…,N     Using ARMS        Sample $\lambda^{\left(k\right)}∼p\left(\lambda |\alpha^{\left(k-1\right)},z^{\left(k-1\right)}\right)$        Sample $\alpha^{\left(k\right)}∼p\left(\alpha |\lambda^{\left(k\right)},z^{\left(k-1\right)}\right)$     For t=1,…,n        If $Y_{t}=L$            For j=1,…,m               Sample $w^{\left(j\right)}∼\mathrm{Po}\left(\frac{\lambda^{\left(k\right)}}{1-\alpha^{\left(k\right)}}\right)\times I_{\left(w^{\left(j\right)}\geq L\right)}$            $z_{t}^{\left(k\right)}:=\left\lceil median\left(w\right)\right\rceil ,w=\left(w^{\left(1\right)},\ldots ,w^{\left(m\right)}\right),$       Else           $z_{t}^{\left(k\right)}:=Y_{t}$Return $\theta ={\left[\theta^{\left(1\right)},\ldots ,\theta^{\left(N\right)}\right]}^{\prime }$ and $z^{\left(N\right)}$.

## 4. Illustration

This section illustrates the procedures proposed above to model CCID(1) right-censored time series in the particular case of Poisson distribution and binomial thinning operation.

### 4.1. Illustration with CensPoINAR(1)

In this section, the performance of the Bayesian approaches previously proposed is illustrated via synthetic data. Thus, realizations with n=100,350,1000 observations of CensPoINAR(1) models were simulated, with parameters θ=(0.2,3) and θ=(0.5,5), considering for each case two levels of censorship, namely 30% and 5%.

For the ABC estimates, we run $N=10^{6}$ replications and choose the pairs (α,λ) corresponding to the 0.1% lower quantile of $d_{S}^{\left(k\right)},$ Equation (7), in total of 1000 values from which the estimates are computed as the mean value. Software R [25] was used to implement the ABC algorithm.

To implement GDA-MMS algorithm we consider the initial values $\theta^{\left(0\right)}=\left(\alpha^{\left(0\right)},\lambda^{\left(0\right)}\right)$ given by the Conditional Least squares estimates of α and λ [24]. The hyper-parameters for the prior distributions of α and λ are the following α∼Beta(a=2,b=2) and λ∼Gamma(c=0.1,d=0.1). In this work, the function *armspp* was used from the package *armspp* [26] in R to sample from the full conditional distributions. Several experiments were carried out to analyse the size that the chain should have in order to be stable and, thus, the number of Gibbs sampler iterations used in this work is *N* = 15,000. Among these, we ignored the first 5000 simulations as burn-in time and, to reduce autocorrelation between MCMC observations, we considered only simulations from every 30 iterations. Therefore, we use a simulated sample with size 323 to obtain the Bayesian estimates. A convergence analysis with the usual diagnostic tests was performed with the package *coda* [27] in R [25].

Table 2 and Table 3 summarize ABC and GDA-MMS results for the several scenarios described above: point estimates, $\hat{\alpha }$ and $\hat{\lambda },$ obtained as sample means, the corresponding bias, standard deviation and the coefficient of variation. The results indicate that the bias tends to decrease for large sample sizes and small censoring rates. The results also indicate that overall ABC presents estimates with smaller bias but larger variability when compared with GDA-MMS.

**Figure 3:**
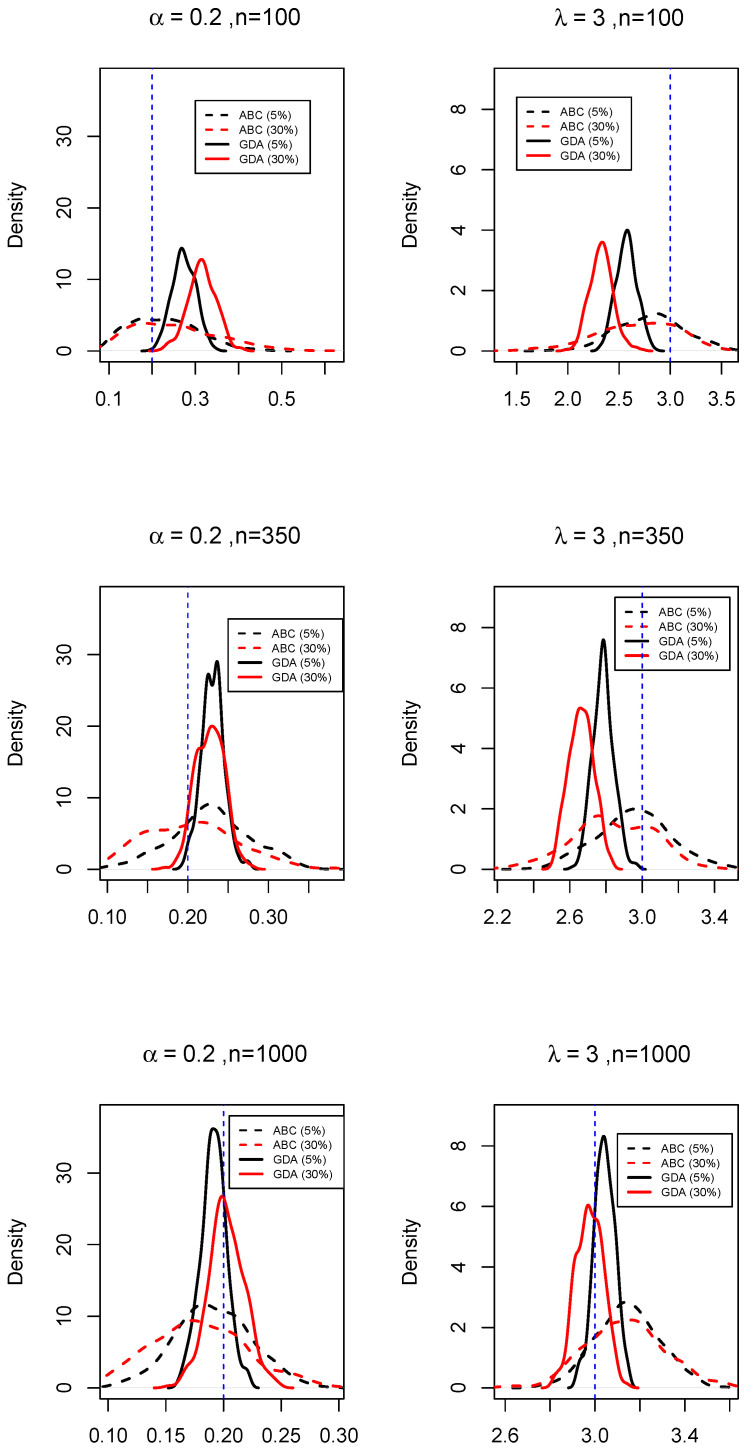
ABC and GDA-MMS posterior densities of the parameters for a realization of 100, 350 and 1000 observations of a CensPoINAR(1) model with θ=(0.2,3), considering two levels of censoring. Note that the scale of *x*-axis of the six plots are different.

**Figure 4:**
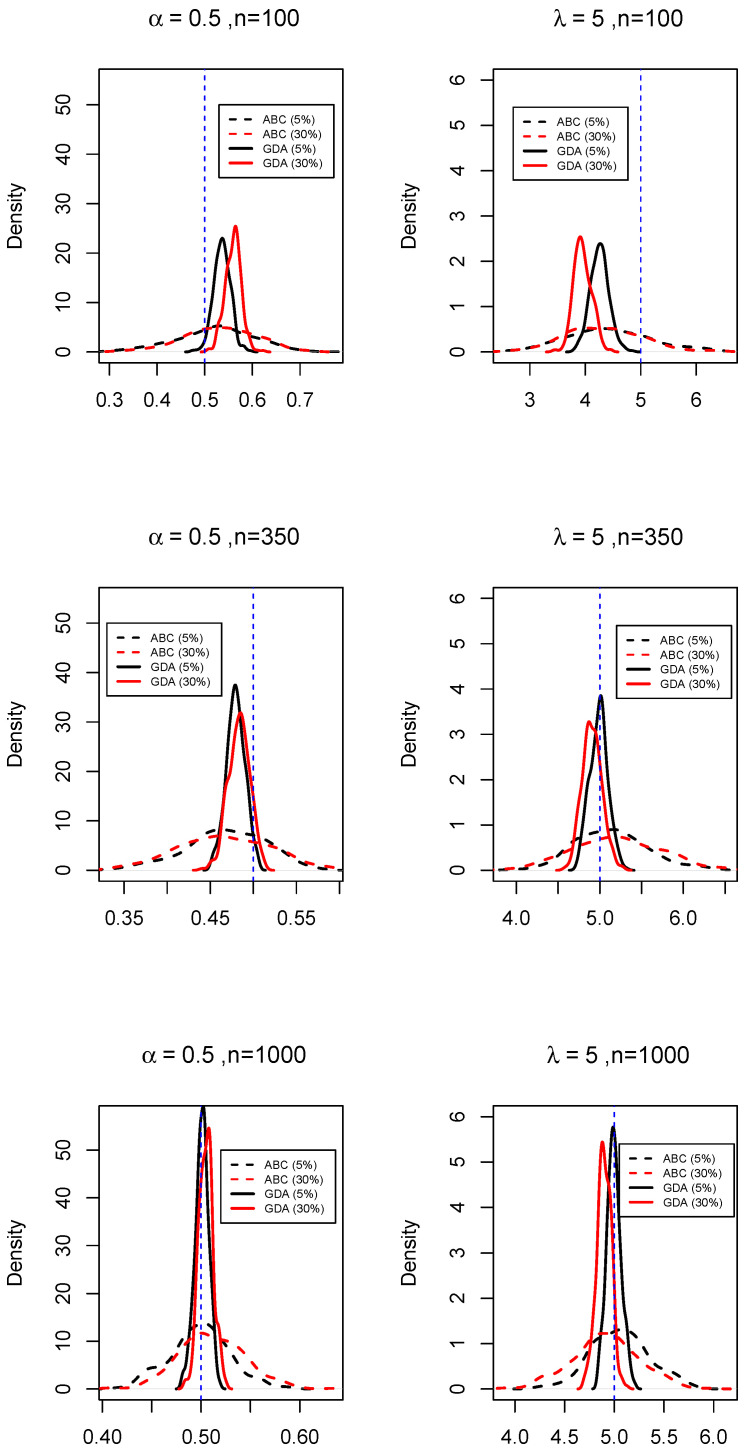
ABC and GDA-MMS posterior densities of the parameters for a realization of 100, 350 and 1000 observations of a CensPoINAR(1) model with θ=(0.5,5), considering two levels of censoring. Note that the scale of *x*-axis of the six plots are different.

Additionally, Figure 3 and Figure 4 represent the corresponding posterior densities. The plots show unimodal and approximately symmetric distributions, with a dispersion that clearly decreases with increasing sample size and smaller censoring rate. The posterior densities indicate that the ABC approach produces posteriors that are flatter but with modes very close to the true value, while the corresponding GDA-MMS approach, despite producing posteriors which are more concentrated also evidence higher bias. However, the behaviour of GDA-MMS estimates varies with the parameters and even the sample sizes. These results are representative of the properties of GDA-MMS estimates across a large number of experiments, not reported here for conciseness.

### 4.2. Simulation Study for GDA-MMS

This section presents the results of a simulation study designed to further analyse the sample properties of GDA-MMS, in particular the bias of the resulting Bayesian estimates.

For that purpose, realizations with sample sizes n=100 and n=350 of CensPoINAR(1) models with parameters θ=(0.2,3) and θ=(0.5,5), are generated, considering two levels of censorship, namely 30% and 5%. To analyse the performance of the procedure, the sample posterior mean, standard deviation and mean squared error were calculated over 50 repetitions.

**Figure 5:**
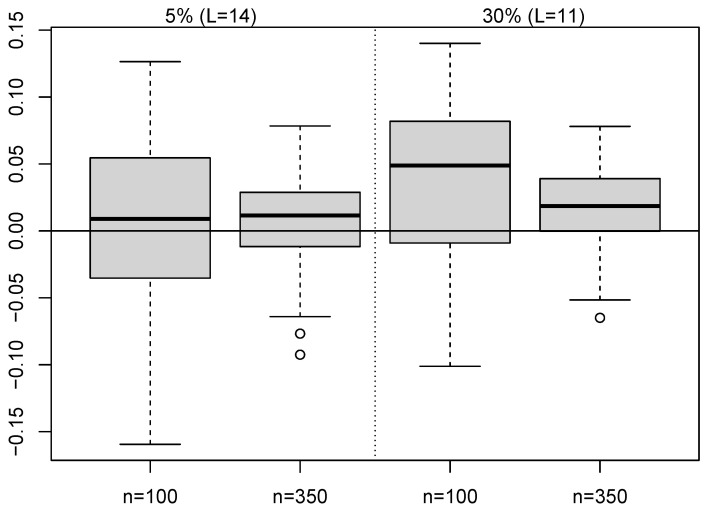
Boxplots of bias for GDA-MMS estimates of α, when θ=(0.5,5).

**Figure 6:**
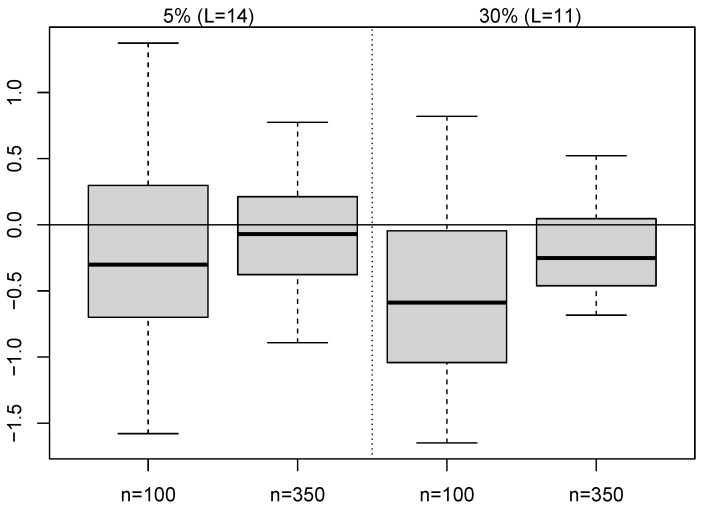
Boxplots of bias for GDA-MMS estimates of λ, when θ=(0.5,5).

Boxplots of the sample bias for the 50 repetitions of GDA-MMS methodology are presented in Figure 5 and Figure 6. The bias increases with the rate of censoring and the variability decreases with the sample size. Furthermore, in general, the estimates for α presents positive sample mean biases, indicating that α is overestimated, whilst the estimates for λ shows negative sample biases, indicating underestimation for λ. Both bias and dispersion seem larger for λ.

Table 4 and Table 5 present the sample posterior measures for $\hat{\alpha }$ and $\hat{\lambda },$ respectively. We can see improvement of the estimation methods performance when the sample size increases. Additionally, the higher the censoring percentage, the worse the behavior of the proposed methods.

## 5. Final Comments

This work approaches the problem of estimating CCID(1) models for time series of counts under censoring from a Bayesian perspective. Two algorithms are proposed: one is based on ABC methodology and the second a Gibbs Data Augmentation modified with multiple sampling. Experiments with synthetic data allow us to conclude that both approaches lead to estimates that present less bias than those obtained neglecting the censoring. Moreover, the GDA-MMS approach allows us to obtain a complete data set, making it a valuable method in other situations such as missing data.

In this study, we focus on the most popular CCID(1) model, the Poisson INAR(1). However, if the data under study present over- or under-dispersion, other CCID(1) models with appropriate distributions for the innovations, such as Generalised Poisson or Negative Binomial, can easily be entertained. Furthermore, one can consider different models for time series of counts under censoring, based on INGARCH models, ([28,29] using a switching mechanism) if they are more suitable to the data set to be modeled. These issues are beyond the scope of this paper and are a topic for a future research project.

## Figures and Tables

**Table 1:** Methods for constructing integer valued AR(1) models with specified marginals $F_{\mu }$ and innovations $e_{t}\overset{iid}{=}F_{\lambda },$ λ=μ(1−α). B(·,·) denotes the beta function.

Marginal Distribution	Random Operator	$g(s|X_{t-1};\alpha )$	Innovations	θ
Poison Po(μ)	binomial thinning	Xt−1sαs(1−α)Xt−1−s	Po(λ)	(μ,α)
Negative binomial NB(μ,ξ)	beta binomial thinning	Xt−1sαs(1−α)Xt−1−s	NB(λ,ξ)	(μ,α,ξ)
Generalised Poisson GP(μ,ξ)	quasi binomial thinning	Xt−1sα(α+s(ξ/μ))s−1 ${\left(1-\alpha -s\left(\xi /\mu \right)\right)}^{X_{t-1}-s}$	GP(λ,ξ)	(μ,α,ξ)

**Table 2:** ABC and GDA-MMS results for parameter α (sample mean, and the corresponding bias, standard deviation and coefficient of variation) for synthetic data generated from CensPoINAR(1) models.

				ABC				GDA-MMS			
α	λ	L	n	$\bar{\hat{\alpha }}$	**Bias(** $\hat{\alpha }$ **)**	**s.d.(** $\hat{\alpha }$ **)**	**CV(** $\hat{\alpha }$ **)**	$\bar{\hat{\alpha }}$	**Bias(** $\hat{\alpha }$ **)**	**s.d.(** $\hat{\alpha }$ **)**	**CV(** $\hat{\alpha }$ **)**
0.2	3	4 (30%)	100	0.2571	0.0571	0.0911	0.3544	0.3155	0.1155	0.0323	0.1024
			350	0.2067	0.0067	0.0579	0.2803	0.2274	0.0274	0.0178	0.0783
			1000	0.1793	−0.0207	0.0398	0.2217	0.2025	0.0025	0.0157	0.0775
0.2	3	6 (5%)	100	0.2268	0.0268	0.0760	0.3350	0.2738	0.0738	0.0270	0.0986
			350	0.2302	0.0302	0.0511	0.2221	0.2309	0.0309	0.0140	0.0606
			1000	0.1931	−0.0069	0.0327	0.1692	0.1915	−0.0085	0.0112	0.0585
0.5	5	11 (30%)	100	0.5304	0.0304	0.0800	0.1508	0.5596	0.0596	0.0170	0.0304
			350	0.4637	−0.0363	0.0535	0.1153	0.4834	−0.0166	0.0124	0.0257
			1000	0.5115	0.0115	0.0320	0.0626	0.5050	0.0050	0.0072	0.0143
0.5	5	14 (5%)	100	0.5230	0.0230	0.0815	0.1559	0.5363	0.0363	0.0175	0.0326
			350	0.4671	−0.0329	0.0461	0.0987	0.4796	−0.0204	0.0107	0.0223
			1000	0.4992	−0.0008	0.0291	0.0584	0.5008	0.0008	0.0070	0.0140

**Table 3:** ABC and GDA-MMS results for the parameter λ (sample mean, and the corresponding bias, standard deviation and coefficient of variation) for synthetic data generated from CensPoINAR(1) models.

				ABC				GDA-MMS			
α	λ	L	n	$\bar{\hat{\lambda }}$	**Bias(** $\hat{\lambda }$ **)**	**s.d.(** $\hat{\lambda }$ **)**	**CV(** $\hat{\lambda }$ **)**	$\bar{\hat{\lambda }}$	**Bias(** $\hat{\lambda }$ **)**	**s.d.(** $\hat{\lambda }$ **)**	**CV(** $\hat{\lambda }$ **)**
0.2	3	4 (30%)	100	2.6623	−0.3377	0.3699	0.1389	2.3265	−0.6735	0.1144	0.0492
			350	2.8530	−0.1470	0.2353	0.0825	2.6639	−0.3361	0.0672	0.0252
			1000	3.1398	0.1398	0.1668	0.0531	2.9757	−0.0243	0.0603	0.0203
0.2	3	6 (5%)	100	2.7918	−0.2082	0.3203	0.1147	2.5719	−0.4281	0.1007	0.0392
			350	2.9507	−0.0493	0.2173	0.0736	2.7846	−0.2154	0.0579	0.0208
			1000	3.1342	0.1342	0.1448	0.0462	3.0417	0.0417	0.0460	0.0151
0.5	5	11 (30%)	100	4.3432	−0.6568	0.7504	0.1728	3.9528	−1.0472	0.1600	0.0405
			350	5.2315	0.2315	0.5265	0.1006	4.9073	−0.0927	0.1177	0.0240
			1000	4.9102	−0.0898	0.3247	0.0661	4.8974	−0.1026	0.0720	0.0147
0.5	5	14 (5%)	100	4.4488	−0.5512	0.7828	0.1760	4.2574	−0.7426	0.1682	0.0395
			350	5.1333	0.1333	0.4286	0.0835	4.9877	-0.0123	0.1088	0.0218
			1000	5.0613	0.0613	0.2826	0.0558	4.9964	−0.0036	0.0708	0.0142

**Table 4:** Sample posterior mean, standard errors (in brackets) and root mean square error for GDA-MMS estimates of α.

α	λ	*L*	*n*	$\bar{\hat{\alpha }}$ (s.e.$\left(\hat{\alpha }\right)$)	RMSE$\left(\hat{\alpha }\right)$
0.2	3	4 (30%)	100	0.2918 (0.0977)	0.1341
			350	0.2385 (0.0698)	0.0797
0.2	3	6 (5%)	100	0.2739 (0.0680)	0.1004
			350	0.2229 (0.0487)	0.0538
0.5	5	11 (30%)	100	0.5404 (0.0632)	0.0750
			350	0.5156 (0.0344)	0.0378
0.5	5	14 (5%)	100	0.5142 (0.0626)	0.0642
			350	0.5066 (0.0386)	0.0392

**Table 5:** Sample posterior mean, standard errors (in brackets) and root mean square error for GDA-MMS estimates of λ.

α	λ	*L*	*n*	$\bar{\hat{\lambda }}$ (s.e.$\left(\hat{\lambda }\right)$)	RMSE$\left(\hat{\lambda }\right)$
0.2	3	4 (30%)	100	2.5283 (0.3077)	0.5632
			350	2.7842 (0.2462)	0.3274
0.2	3	6 (5%)	100	2.6814 (0.2984)	0.4365
			350	2.8934 (0.1843)	0.2129
0.5	5	11 (30%)	100	4.4861 (0.6710)	0.8452
			350	4.7976 (0.3357)	0.3920
0.5	5	14 (5%)	100	4.7593 (0.6391)	0.6829
			350	4.9229 (0.4177)	0.4248
